# Mitochondrial-targeted therapy for osteoarthritis: Challenges and opportunities from basic research to clinical translation

**DOI:** 10.3389/fimmu.2026.1696120

**Published:** 2026-02-20

**Authors:** Song-Ou Zhang, Zhi-Qian Gu, Jian Ruan, Jian Zhang, Hong Chen

**Affiliations:** 1 School of medicine, Ningbo University, Ningbo, Zhejiang, China; 2 Department of Hand Surgery, Ningbo No.6 Hospital, Ningbo, Zhejiang, China

**Keywords:** clinical translation, drug delivery, mitochondrial dysfunction, mitophagy, osteoarthritis, oxidative stress, targeted therapy

## Abstract

Osteoarthritis is a high-burden degenerative joint disease. Existing therapies only alleviate symptoms but fail to halt disease progression. Studies have identified mitochondrial dysfunction as a core driver of cartilage degeneration in OA. Key mechanisms include mitochondrial reactive oxygen species bursts that activate inflammatory and cell death pathways; imbalances in mitochondrial dynamics leading to fragmentation; autophagy defects causing damage accumulation; and reduced biogenesis coupled with hyperglycolysis, which exacerbates the energy crisis. Collectively, these processes accelerate cartilage destruction. This review focuses on mitochondrial-targeted therapeutic strategies, including antioxidants, dynamics regulators to restore fission-fusion balance, autophagy activators to clear damaged mitochondria, biogenesis enhancers to improve metabolism, and the emerging approach of mitochondrial transplantation to directly replenish functional units. While preclinical studies have demonstrated that these strategies can significantly slow cartilage degeneration, their clinical translation data in OA remain limited. Substantial, translational efforts face three major challenges: drug delivery barriers, disease heterogeneity, and limitations of animal models. Future work will require the development of intelligent delivery systems, patient stratification, and humanized models to promote clinical translation.

## 1 Introduction

Osteoarthritis (OA) is a chronic, degenerative joint disease characterized by progressive degeneration of articular cartilage, subchondral bone sclerosis, osteophyte formation, synovial inflammation, and joint capsule hypertrophy (1, 2). It is the most common joint disease worldwide, severely impacting patients’ quality of life and imposing a heavy socioeconomic burden. Epidemiological data show that the prevalence of OA increases significantly with age, reaching 10%-15% in people over 60 years old, with a higher incidence in women than in men (3–5). With the aging of the global population and rising obesity rates, the prevalence of OA is expected to continue rising (6). The core pathological process of OA begins with the degradation of articular cartilage, manifested by an imbalance between the synthesis and catabolism of the cartilage extracellular matrix (primarily collagen II and proteoglycans). This imbalance ultimately leads to cartilage thinning and rupture. A concomitant synovial inflammatory response releases proinflammatory cytokines and proteases, further exacerbating cartilage destruction (7). Abnormal subchondral bone remodeling (sclerosis, cyst formation) and marginal osteophyte proliferation are also hallmark changes of OA. These pathological changes collectively lead to joint pain, stiffness, limited mobility, and functional impairment (8). OA not only causes tremendous physical and mental suffering for patients, but also, due to its high incidence, chronic course, and impact on the workforce, leads to soaring medical costs, lost productivity, and decreased quality of life, making it a major global public health challenge (5).

Current OA treatment strategies primarily focus on relieving symptoms and improving function, but a definitive cure that can effectively slow or reverse disease progression remains lacking. Analgesics such as nonsteroidal anti-inflammatory drugs and acetaminophen are commonly used for symptomatic treatment. However, long-term use is often associated with severe gastrointestinal, cardiovascular, and renal side effects. These drugs provide only partial pain relief and fail to prevent cartilage destruction. Opioid use is strictly restricted due to the risks of addiction and other adverse reactions. Intra-articular injections of glucocorticoids or hyaluronic acid can improve symptoms in the short term, but their efficacy is limited, and their role in modifying disease progression is unclear (9–11). The development of highly anticipated disease-modifying osteoarthritis drugs has been slow, and no widely recognized effective drugs have been approved to date (12, 13). For patients with end-stage OA, joint replacement surgery (such as knee and hip arthroplasty) is an effective, definitive solution that significantly reduces pain and restores function. However, surgery is inherently invasive, costly, and carries the risk of complications. Furthermore, prostheses have a limited lifespan, making this option particularly unsuitable for young or early-stage patients (14, 15). Therefore, the development of novel therapeutic strategies that can target the core pathological mechanisms of OA to safely and effectively delay or prevent disease progression is urgently needed.

Given the multifactorial pathogenesis of OA, various signaling pathways—including cellular senescence, mechanical signaling, and the Wnt pathway—have been extensively studied as potential therapeutic targets. While these pathways are undoubtedly important, mitochondrial dysfunction has emerged as an upstream regulator and central hub that initiates and integrates these pathological processes. In contrast to the well-reviewed pathways above, systematic reviews focusing on mitochondria as a therapeutic target in OA remain relatively scarce. This review aims to address this gap by positioning mitochondrial dysfunction as a pivotal mechanism, thereby justifying our focused investigation into its potential for therapeutic intervention. Mitochondria, the “energy factories” of eukaryotic cells, have the core function of producing adenosine triphosphate (ATP) through oxidative phosphorylation, providing energy for cellular activity. However, their role extends far beyond energy production. Mitochondria also participate in the generation and removal of reactive oxygen species (ROS), maintain intracellular calcium homeostasis, initiate specific apoptotic pathways, and synthesize a variety of metabolic intermediates (16, 17). In articular cartilage, chondrocyte survival and function are highly dependent on healthy mitochondria to maintain energy supply in hypoxic environments, synthesize the extracellular matrix, and resist various stresses (18).

Increasing evidence indicates that mitochondrial dysfunction is an early and key driver of OA, particularly chondrocyte degeneration (19, 20). Under OA pathological conditions, multiple factors (such as aging, mechanical stress, and inflammatory factors) can lead to a series of mitochondrial dysfunctions. These include an energy crisis with reduced ATP production, which impairs chondrocyte anabolic capacity and stress resistance (21). Oxidative stress, characterized by excessive mitochondrial ROS (mtROS) production, overwhelms the cell’s antioxidant defenses (22, 23). Excessive mtROS not only directly damages mitochondrial DNA, proteins, and lipids, further deteriorating mitochondrial function, but also activates inflammatory signaling pathways. This induces chondrocyte senescence and stimulates the expression of matrix metalloproteinases (MMPs) and a disintegrin and metalloproteinase with thrombospondin motifs, accelerating cartilage matrix degradation (24–26). Calcium homeostasis is also disrupted. As mitochondria participate in intracellular calcium buffering, their dysfunction can lead to intracellular calcium overload, activating calcium-dependent proteases, disrupting cellular structure, and promoting apoptosis. An imbalance in mitochondrial dynamics, with persistent fission exceeding fusion, leads to mitochondrial fragmentation (27). These fragmented mitochondria are functionally impaired, more susceptible to ROS production, and prone to triggering apoptosis. Furthermore, mitophagy, the selective clearance of damaged mitochondria, is impaired in OA, leading to the accumulation of damaged organelles and a vicious cycle of dysfunction (28). These interrelated events collectively constitute the core pathological basis of OA, driving chondrocyte death, reduced matrix synthesis, and increased degradation. This makes mitochondria an attractive new target for OA treatment (29, 30).

This review aims to systematically examine the basic research evidence for mitochondrial-targeted therapies for OA, providing an in-depth analysis of potential mechanisms of action, key targets, and candidate drugs/strategies. We will focus on analyzing the core challenges currently facing the translation of basic research findings into clinical applications. Furthermore, we will explore potential opportunities and future directions for overcoming these challenges, aiming to provide theoretical insights and practical guidance for advancing mitochondrial-targeted therapies as effective treatments for OA.

## 2 Mitochondrial dysfunction in OA

The multifaceted mitochondrial dysfunction in OA is not a series of isolated events but an interconnected pathological network. While oxidative stress is a pivotal initiating and amplifying factor, it engages in reciprocal interactions with other key processes—including dynamics imbalance, impaired mitophagy, and compromised biogenesis—creating a self-perpetuating cycle that drives chondrocyte failure and cartilage degradation. The following sections detail these core mechanisms and their synergistic interplay.

### 2.1 The central role of mitochondrial oxidative stress

Numerous studies have demonstrated significant overproduction of mtROS in osteoarthritic joint tissues, particularly cartilage and synovium (31, 32). This can be detected using various methods, such as mitochondria-specific fluorescent probes that show enhanced signals in OA chondrocytes or animal models, as well as elevated levels of mitochondrial oxidative damage markers (33–35). This excess mtROS is a core driver of OA pathology, not a harmless byproduct (36). First, mtROS is a potent inflammatory activator that directly activates the NOD-like receptor family, pyrin domain containing 3 (NLRP3) inflammasome, leading to caspase-1 activation and the maturation and release of proinflammatory cytokines interleukin-1 beta (IL-1β) and interleukin-18 (IL-18), thereby amplifying joint inflammation (37, 38). Notably, the resultant inflammatory mediators can, in turn, further elevate mtROS production, establishing a feed-forward loop. Moreover, this sustained oxidative and inflammatory milieu directly inflicts damage on mitochondrial structure and function, serving as a critical upstream trigger for the dynamics imbalance and mitophagy impairment described below.​ Second, mtROS induces chondrocyte senescence by activating signaling pathways such as p38 mitogen-activated protein kinase (MAPK) and nuclear factor-kappa B (NF-κB), manifested by increased Senescence-Associated β-Galactosidase (SA-β-gal) activity and upregulated p16/p21 expression. The senescence-associated secretory phenotype secreted by these cells further disrupts the microenvironment (39, 40). Third, mtROS upregulates the expression of matrix-degrading enzymes, accelerating the breakdown of the cartilage matrix (41). Finally, mtROS induces the opening of the mitochondrial permeability transition pore (mPTP), promotes the release of cytochrome c (Cyt c), activates the caspase cascade, and triggers chondrocyte apoptosis (42). mtROS-mediated NLRP3 inflammasome activation is also a key pathway for pyroptosis, leading to lytic cell death and the release of inflammatory media, forming a vicious cycle (43, 44).

### 2.2 Mitochondrial dynamics imbalance

In the OA pathological state, chondrocytes commonly exhibit a mitochondrial dynamics imbalance, characterized by excessive fission and insufficient fusion, leading to a fragmented mitochondrial network (45). This fragmentation can be observed via electron microscopy as reduced mitochondrial size, increased number, and irregular structure, or by fluorescence microscopy as a transformation from elongated tubular networks to short, punctate particles (46, 47). Mitochondrial fragmentation has multiple negative impacts: (1) Decreased energy production: Fragmented mitochondria have impaired assembly of electron transport chain complexes, reducing oxidative phosphorylation (OXPHOS) efficiency and ATP synthesis, which weakens the energy foundation for maintaining matrix homeostasis. (2) Increased ROS production: Impaired electron transport in fragmented mitochondria leads to increased electron leakage and excessive mtROS production, exacerbating oxidative stress. (3) Promotion of apoptosis: Excessive fission is accompanied by mitochondrial outer membrane permeabilization (MOMP), promoting the release of pro-apoptotic factors into the cytosol and activating apoptosis. The key protein driving mitochondrial fission is dynamin-related protein 1 (Drp1), whose activity is regulated by post-translational modifications such as phosphorylation and sumoylation (48–50). Studies have shown that Drp1 expression is increased in OA chondrocytes, while the expression of fusion proteins is downregulated (51, 52). Therefore, regulating these dynamics proteins has become a potential therapeutic target.

### 2.3 Dysregulation of mitophagy

The accumulation of fragmented and dysfunctional mitochondria resulting from excessive fission imposes a critical burden on the mitophagic clearance system. Mitophagy is an autophagic process that selectively eliminates damaged or dysfunctional mitochondria and is crucial for maintaining mitochondrial quality control and cellular homeostasis (53, 54). The PINK1-Parkin pathway is a major mechanism: decreased membrane potential in damaged mitochondria stabilizes PINK1 on the outer membrane, which recruits and phosphorylates Parkin. Parkin ubiquitinates mitochondrial outer membrane proteins, recruiting autophagy receptors and autophagosomes (LC3-II)to encapsulate and degrade the damaged organelle (54, 55). In OA, this quality control mechanism is impaired. OA chondrocytes show decreased expression or activity of PINK1 and Parkin (56). Key autophagy-related protein levels are abnormal, autophagic flux assays reveal suppressed activity, and damaged mitochondria accumulate (57, 58). Impaired mitophagy prevents the clearance of dysfunctional mitochondria, which continuously produce excessive mtROS and release pro-apoptotic factors. This creates a vicious cycle of “damage accumulation and functional deterioration,” exacerbating oxidative stress, energy crisis, and cellular dysfunction, wherein failed clearance exacerbates the very oxidative stress and dynamics imbalance that generated the damaged organelles in the first place, ultimately promoting chondrocyte death and matrix degradation (59). Therefore, enhancing mitophagy is a promising therapeutic strategy. Studies have shown that upregulating PINK1/Parkin using inducers or genetic modulation can mitigate mitochondrial damage, reduce oxidative stress, and protect chondrocytes in OA models (60–62). However, this strategy carries potential risks, as excessive or uncontrolled mitophagy may lead to the excessive clearance of healthy mitochondria, also impairing cellular function. Future research should focus on developing methods to precisely and appropriately regulate mitophagy levels.

### 2.4 Mitochondrial biogenesis and metabolic reprogramming

Confronted with the compounded deficits in quality control and an accumulating burden of damaged mitochondria, the compensatory pathway of generating new mitochondria is also suppressed.​ Mitochondrial biogenesis is critical for maintaining a sufficient population of functional mitochondria and is primarily regulated by PGC-1α and its downstream factors (nuclear respiratory factor 1 [NRF1], nuclear respiratory factor 2 [NRF2], and mitochondrial transcription factor A [TFAM]) (63–65). PGC-1α is a master regulator, activating the transcription of nuclear-encoded mitochondrial genes (66). In OA chondrocytes, PGC-1α expression is significantly downregulated, accompanied by decreased expression of its target genes and a reduced mitochondrial DNA (mtDNA) copy number, indicating impaired biogenesis capacity (67). As a damage-associated molecular pattern, extruded mtDNA can engage intracellular sensors—such as the cGAS-STING pathway—triggering sterile inflammatory responses that exacerbate the OA phenotype. This process, along with the diminished mitochondrial pool, exacerbates cellular energy insufficiency. This leads to a decrease in functional mitochondria, exacerbating energy insufficiency.

**Figure 1:**
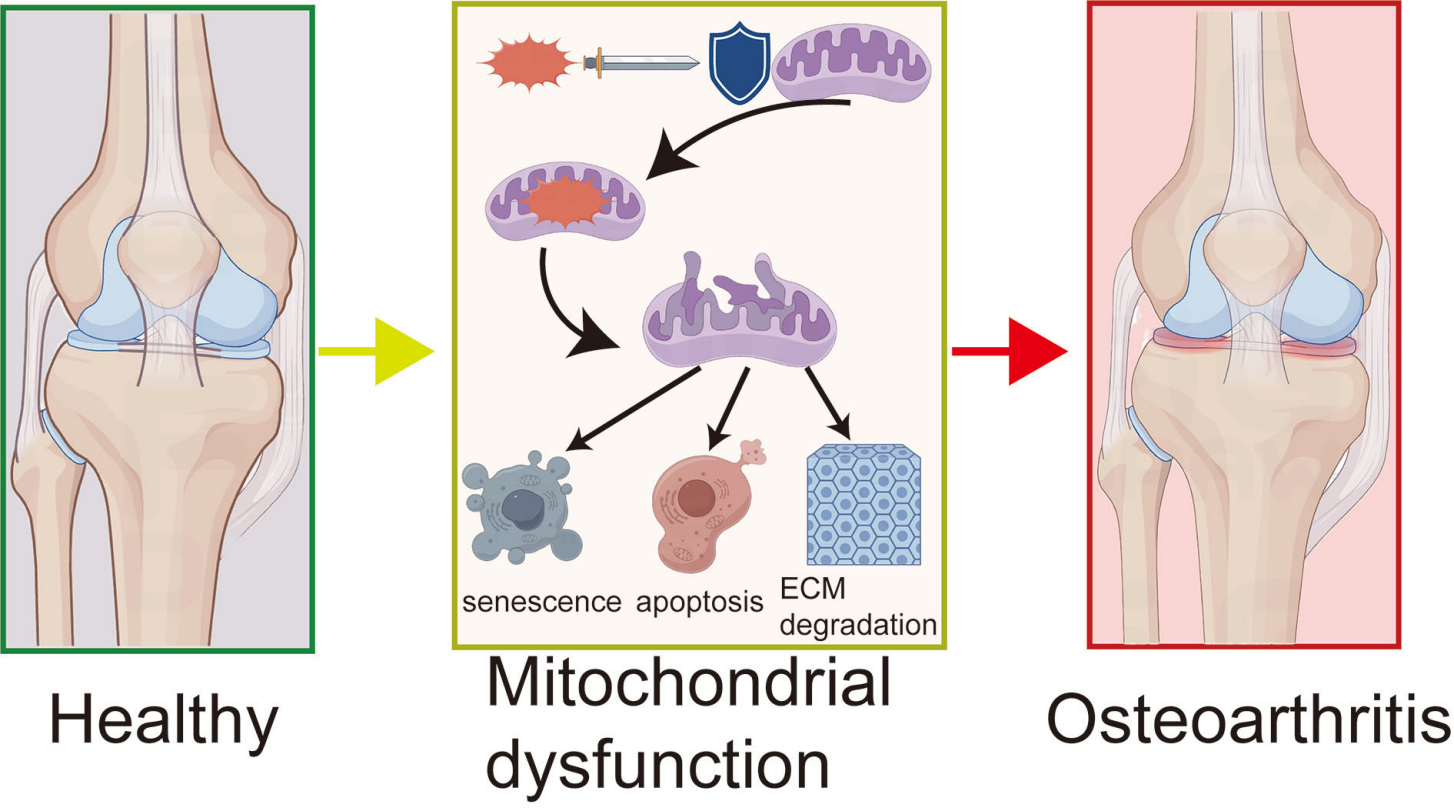
The mechanism of mitochondrial dysfunction in OA.

The collective outcome of the dysfunctions described above—energy crisis, oxidative damage, and a diminished functional mitochondrial pool—precipitates a fundamental shift in cellular metabolism.​ OA chondrocytes undergo significant metabolic reprogramming. To cope with the energy crisis and hypoxic microenvironment, they switch from efficient oxidative phosphorylation to a less efficient glycolytic pathway (36, 68). This shift is manifested by upregulated expression of key glycolytic enzymes (e.g., hexokinase 2 [HK2], pyruvate kinase isozyme M2 [PKM2], and lactate dehydrogenase A [LDHA]) and increased lactate production (69–73). While glycolysis can rapidly provide ATP in the short term, its long-term consequences exacerbate energy and substrate shortages for extracellular matrix synthesis and may affect cellular pH and signaling. However, excessive glycolysis leads to lactic acid buildup and a localized acidic microenvironment. This acidic microenvironment impairs the production of chondrocyte matrix and may exacerbate cartilage degeneration in osteoarthritis (74). Furthermore, accumulated lactic acid, as a precursor to epigenetic modifications, specifically promotes lactation of histone H3 at position 18 in fibrochondrocytes, thereby promoting transcriptional activation of key fibrosis-related genes such as Itga6, Cxcl10, and Parp16 (75). Mitochondrial dysfunction is a key driver of this metabolic reprogramming. Therefore, enhancing mitochondrial biogenesis by activating the peroxisome proliferator-activated receptor gamma coactivator 1 alpha pathway or regulating key metabolic enzymes to improve OXPHOS efficiency or moderately reverse abnormal glycolysis has emerged as a potential therapeutic approach for OA, aiming to restore chondrocyte energy homeostasis and anabolic function (**Figure 1**).

This figure reveals the process of OA development: ROS attack leads to mitochondrial dysfunction, which in turn triggers chondrocyte senescence, apoptosis, and ECM degradation, ultimately causing articular cartilage destruction and OA. The diagram shows a comparison from left to right of healthy joints, the core mechanisms of the disease, and OA-affected joints.

## 3 Mitochondrial-targeted OA treatment strategies

### 3.1 Mitochondrial-targeted antioxidant therapy

Mitochondrial-targeted antioxidant therapy aims to precisely neutralize mtROS and mitigate oxidative stress-induced chondrocyte damage. Its key advantage is overcoming the limitation of traditional antioxidants, which fail to effectively accumulate in mitochondria. Strategies include: (1) designing molecular carriers to deliver antioxidant groups directly to mitochondria; (2) modulating endogenous antioxidant enzymes to enhance mROS scavenging capacity; and (3) using advanced delivery systems to improve intra-articular drug bioavailability.

Representative agents like MitoQ (a mitochondria-targeted coenzyme Q10 derivative) and SS-31 (elamipretide, a cell-penetrating peptide targeting the inner mitochondrial membrane) have shown significant protective effects in preclinical studies. Grishko et al. found that hyaluronic acid pretreatment significantly reduced mtDNA damage (reduced 8-hydroxy-2’-deoxyguanosine [8-OHdG] levels) in human chondrocytes under oxidative stress, enhanced mtDNA repair capacity, maintained ATP levels, and reduced apoptosis, suggesting protection via ameliorating mitochondrial oxidative stress (76). Farnaghi et al. demonstrated that combining mitochondria-targeted antioxidants with statins in a hypercholesterolemia-induced OA model effectively inhibited ROS overload, alleviated mitochondrial dysfunction, and delayed cartilage matrix degradation and osteophyte formation (77). These effects were linked to the inhibition of mtROS-activated NLRP3 inflammasome and IL-1β release (77). Natural molecules such as melatonin also show potential. Zhang et al. demonstrated that melatonin, by activating the sirtuin 1 (SIRT1)/superoxide dismutase 2 (SOD2) axis, restored mitochondrial membrane potential (Δψm) in OA chondrocytes, reduced mtROS levels, promoted ATP synthesis, and enhanced ECM synthesis (78). To overcome rapid joint clearance and poor cartilage permeability, novel delivery systems are being developed. Da et al. constructed tannic acid nanoparticles loaded with metformin (Met@TA NPs). This system leveraged TA’s antioxidant activity and Met’s mitochondrial protection to enable intelligent drug release in the acidic OA microenvironment. The NPs efficiently scavenged ROS, restored Δψm, improved mitochondrial quality via promoted mitophagy, and inhibited chondrocyte senescence and matrix degradation (79). However, challenges remain. Hines et al. found that under non-stress conditions, vitamin E increased mitochondrial biogenesis but also increased free radical production. Overexpression of glutathione peroxidase 4 (GPX4) reduced oxidative damage markers but disrupted the physiological balance of mitochondrial redox homeostasis in aged mice, suggesting that antioxidant interventions require careful regulation to avoid disrupting normal metabolism (80). Furthermore, while molecules like SS-31 have demonstrated safety in clinical trials for other diseases, their clinical translation in OA is limited by intra-articular delivery efficiency, patient heterogeneity, and a lack of long-term efficacy validation. Future approaches will require intelligent responsive nanocarriers and biomarker-based patient stratification to optimize the therapeutic window and facilitate clinical evaluation (78, 79). A critical concept underpinning these future approaches is the “redox window” for therapeutic intervention.​ This refers to the optimal concentration range of antioxidants that effectively suppresses pathological mtROS bursts to alleviate damage, while still preserving the physiological levels of ROS required for normal cellular signaling. Defining this therapeutic threshold is paramount. Excessively aggressive antioxidant strategies risk collapsing the physiological ROS gradient across the mitochondrial membrane, potentially impairing vital signaling and metabolic adaptation. Therefore, the goal of next-generation mitochondrial-targeted therapies is not to eliminate mtROS entirely, but to modulate it back within a physiological “window,” thereby restoring redox homeostasis without causing reductive stress. The development of smart, conditionally activated antioxidants and precise patient stratification will be crucial to achieve this delicate balance.

### 3.2 Therapeutic strategies for regulating mitochondrial dynamics

Mitochondrial dynamics, specifically the balance between fusion and fission, is key for maintaining chondrocyte energy homeostasis and survival. The mitochondrial fragmentation resulting from its dysregulation is a core driver of chondrocyte dysfunction in OA. Modulating dynamics, particularly by inhibiting excessive fission or promoting fusion, is a promising therapeutic strategy.

Studies have shown that Drp1 is a core executor of fission, and its activity is regulated by phosphorylation: phosphorylation at Ser616 promotes mitochondrial recruitment and activates fission, while phosphorylation at Ser637 inhibits its activity (81, 82). In OA, proinflammatory cytokines upregulate Drp1 expression and induce Ser616 phosphorylation by activating the ERK1/2 pathway, leading to excessive fission and fragmentation (83). This fragmentation impairs OXPHOS efficiency, increases mtROS leakage, and promotes Cyt c release, triggering apoptosis. Ansari et al. demonstrated that the Drp1 inhibitor Mdivi-1 effectively inhibited IL-1β-induced mitochondrial fragmentation, mtROS burst, and caspase-3 activation, reducing chondrocyte apoptosis (83). Zhang et al. found that mechanical stress has a bidirectional regulatory effect: moderate stress enhances mitochondrial network stability by upregulating fusion proteins (mitofusin 1/2 [MFN1/2] and optic atrophy 1 [OPA1]) and promoting Drp1 translocation, resisting IL-1β-induced damage; excessive stress exacerbates fission (84). This finding provides a basis for customized physical interventions.

Enhancing fusion is another key strategy. Fibroblast growth factor 18 (FGF18) is a potential treatment for early osteoarthritis. Studies have found that FGF18 can play a cartilage-protective role by increasing the fusion and division of chondrocytes in an inflammatory environment (85). TANK-binding kinase 1 (TBK1) is a key regulator of Drp1 activity and mitophagy coupling (86, 87). Hu et al. revealed that TBK1 expression is downregulated in OA cartilage, leading to insufficient Drp1 Ser637 phosphorylation and driving excessive fission. TBK1 overexpression phosphorylates Drp1 at Ser637, inhibiting its fission function and promoting PINK1/Parkin mediated mitophagy (88). In TNF-α-stimulated chondrocytes, TBK1 overexpression reversed fragmentation, restored membrane potential, reduced mtROS, and inhibited apoptosis (88). In a rat surgical OA model, intra-articular injection of a TBK1-encoding lentivirus reduced cartilage degeneration and osteophyte formation by remodeling the mitochondrial network and enhancing autophagic flux (88). Growth factors also participate in regulation. Du et al. found that transforming growth factor (TGF)-β3 activates AMP-activated protein kinase (AMPK) via the Smad3 pathway, inducing mitochondrial fission. Under physiological conditions, this fission may facilitate the clearance of damaged mitochondria and network renewal, suggesting context-dependent regulation by TGF-β3 (89). Furthermore, transplanting mitochondria derived from mesenchymal stem cells can enhance mitochondrial fusion, thereby restoring the energy state of chondrocytes (90).

Novel nano-delivery systems offer advantages. Li et al. developed gold@CeO_2_ core-shell nanozymes (Au@CeO_2_ YSNs) that neutralize mtROS and inhibit ERK1/2-mediated Drp1 Ser616 phosphorylation, restoring mitochondrial homeostasis. Nanoparticles loaded with the chondrogenic peptide CK2.1 (Au@CeO_2_-CK2.1) released the drug under near-infrared light, improving mitochondrial function and enhancing chondrocyte responsiveness to anabolic stimuli by blocking the ERK1/2-Drp1 axis, effectively promoting cartilage regeneration in a mouse OA model (91). He et al. suggest that sympathetic nerve activation upregulates genes involved in fission, fusion, and autophagy, leading to global hyperkinetics and metabolic imbalance, providing a new avenue for intervention via receptor antagonists (92). Clinical translation remains challenging. Individual differences in mitochondrial genetic background may affect efficacy. Fajardo et al. found that individuals carrying mitochondrial haplogroup HV showed faster leukocyte telomere shortening and a significantly higher incidence of OA than carriers of the JT haplogroup, suggesting mitochondrial genetic variation may determine the applicable population for dynamics interventions by affecting aging and oxidative stress sensitivity (93).

### 3.3 Interventions to regulate mitophagy

Mitophagy, a key quality control mechanism, plays a dual role in maintaining chondrocyte homeostasis: moderate activation eliminates dysfunctional mitochondria and interrupts the ROS-inflammation-cell death cycle, whereas defective or excessive autophagy accelerates OA progression. Targeting the mitophagy pathway is an emerging strategy, focusing on receptor-mediated pathways such as PTEN-induced kinase 1 (PINK1)/Parkin, Bcl-2/adenovirus E1B 19 kDa protein-interacting protein 3 (BNIP3)/BNIP3-like (NIX), and FUN14 domain containing 1 (FUNDC1) (94). In OA, proinflammatory cytokines IL-1β and TNF-α contribute to damaged mitochondrial accumulation by inhibiting SIRT3 expression (95) or interfering with the PINK1/Parkin cascade (83). Ansari et al. found that Parkin ubiquitinates mitochondrial proteins and recruits p62/sequestosome 1 (SQSTM1) to clear depolarized mitochondria, reducing the apoptosis rate of OA chondrocytes (96). Shin’s research suggests that PINK1 overexpression may exacerbate cartilage degeneration through excessive autophagy, highlighting the need for fine-grained regulation (97).

Pharmacological interventions focus on natural compounds and small molecules. Curcumin slows cartilage degeneration in a rat OA model by activating the AMPK/PINK1/Parkin axis, restoring Δψm, and reducing mtROS (60). Melatonin enhances PINK1/Parkin-mediated autophagic flux through SIRT1/SOD2 signaling; its sustained-release delivery system improves ECM synthesis in rat cartilage (78). Met@TA NPs utilize acidic microenvironment-responsive release to scavenge ROS and promote mitophagy, restoring membrane potential *in vitro* (79). β-Hydroxybutyrate (β-OHB), a ketone metabolite, activates the AMPK/PINK1 pathway via the hydroxycarboxylic acid receptor 2 (HCAR2) receptor, reversing T-2 toxin-induced autophagy defects while inhibiting the NLRP3 inflammasome (61). Baicalin inhibits the PI3K/Akt/mTOR axis and upregulates PINK1/Parkin and Drp1 expression, enhancing mitophagy in IL-1β-stimulated chondrocytes (98). Beyond natural compounds, repurposed clinical drugs show promise. The classic thiol antioxidant tiopronin has been demonstrated to promote extracellular matrix anabolism and alleviate ROS in OA models by activating the Bnip3-Pink1-Parkin signaling pathway, presenting a novel therapeutic application for a well-established drug (99). Similarly, the small molecule MK8722 alleviates OA cartilage degeneration by activating Sestrin 2, which subsequently upregulates BNIP3 and NRF2 to promote mitophagy, thereby reducing oxidative stress and inhibiting chondrocyte ferroptosis (100).

Gene and biological therapies enable long-term interventions. Hu et al. showed that TBK1 overexpression phosphorylates Drp1 at Ser637, inhibiting excessive fission and enhancing PINK1/Parkin-mediated mitophagy; intra-articular TBK1 lentivirus injection reduced cartilage degradation in mice (88). FUNDC1, a hypoxia-sensitive mitophagy receptor, was shown to be activated by phosphofructokinase P (PFKP)-dependent dephosphorylation. KD025 (a ROCK2 inhibitor) enhanced the FUNDC1-PFKP interaction and improved subchondral bone sclerosis in a mouse OA model (101). Engineered exosome technology enables targeted delivery: human urinary stem cell-derived exosomes loaded with miR-140 (hUSCs-140-Exos) inhibit CAPN1 to stabilize PINK1, penetrate cartilage, and restore autophagic flux (102). Clustered regularly interspaced short palindromic repeats (CRISPR)/CRISPR-associated protein 9 (Cas9) nanotrailers carrying the FOXO3 gene (FoxO3-NETT@SMs) enhance autophagy and inhibit apoptosis by regulating the PINK1/Parkin pathway, offering a tool for personalized treatment (103).

Physical intervention and metabolic regulation expand non-drug approaches. Focused low-intensity pulsed ultrasound promotes autophagosome-lysosome fusion via the phosphoglycerate mutase family member 5 (PGAM5)/FUN14 domain containing 1 (FUNDC1) pathway, increasing the LC3-II/LC3-I ratio in chondrocytes (104). Electroacupuncture upregulates PINK1/Parkin to enhance autophagy, improving joint function in a rabbit OA model (105). Notably, short-term fasting maintains mitochondrial homeostasis by activating optineurin (OPTN)-dependent mitophagy, while OPTN knockout mice exhibit autophagy defects and accelerated OA (106). α-Ketoglutarate, a tricarboxylic acid cycle (TCA) cycle metabolite, enhances autophagy by activating AMPK/mTOR, increasing collagen II expression in aged rat cartilage (28). In summary, regulating mitophagy holds great promise for OA treatment, but breakthroughs in mechanistic depth, delivery precision, and clinical validation are needed to advance its translation. Excessive mitophagy can then be harmful to other tissues; for example, excessive mitophagy may be associated with tumor development (107). Therefore, organ or tissue selectivity for mitophagy-based therapies is necessary.

### 3.4 Promoting mitochondrial biogenesis and metabolic regulation

**Figure 2:**
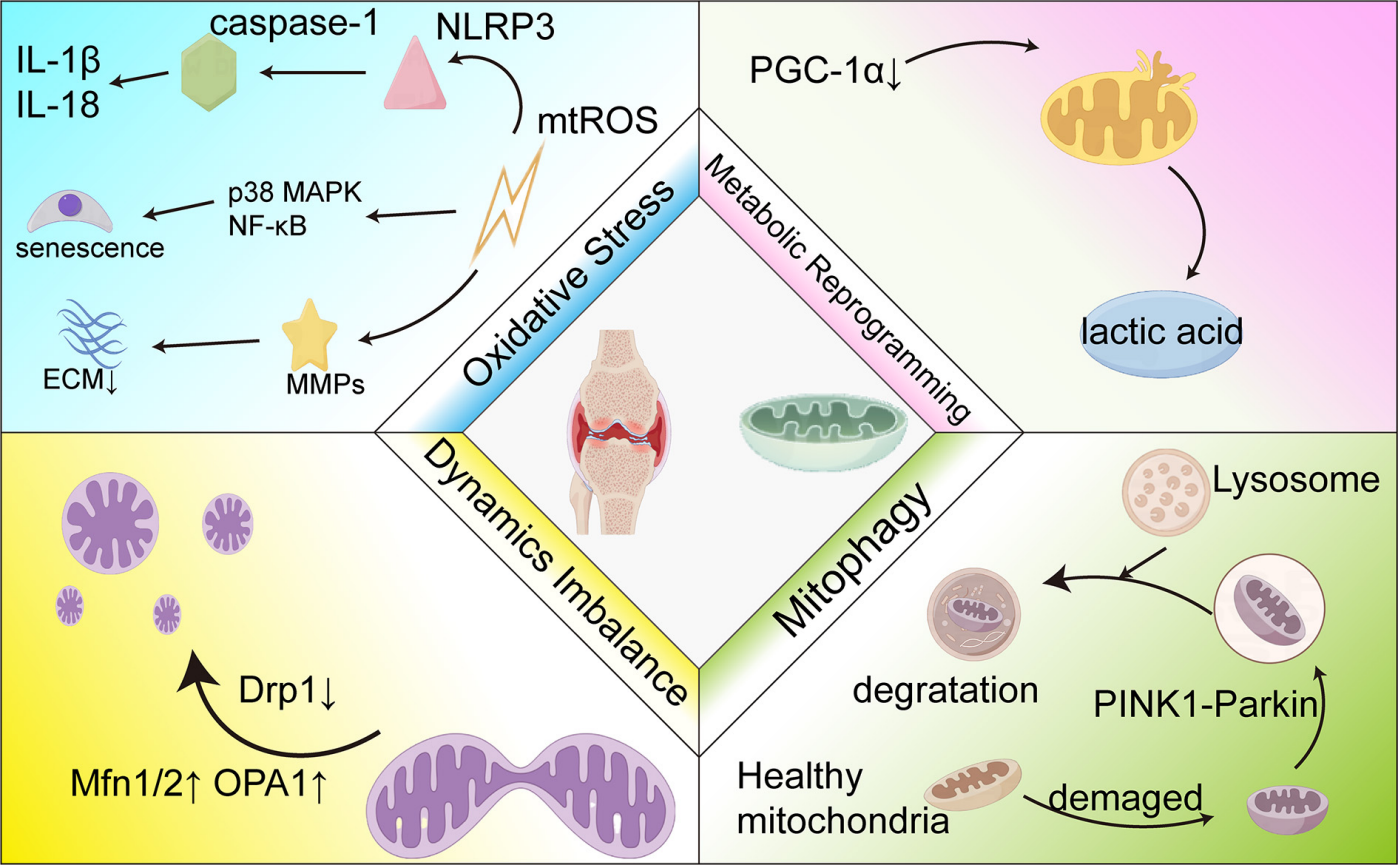
Mitochondrial therapy as a target in OA treatment.

Imperfections in mitochondrial biogenesis and metabolic reprogramming are core pathological features of the chondrocyte energy crisis in OA. Wang et al. found that TFAM expression was decreased in OA patient cartilage, and the mtDNA/nuclear DNA (nDNA) ratio negatively correlated with the degree of cartilage degeneration (108). While metabolic reprogramming to glycolysis maintains short-term ATP supply, it leads to a long-term shortage of UDP-glucosamine (a substrate for matrix synthesis) and lactate accumulation, which can induce Δψm collapse. Activating the PGC-1α signaling axis can effectively reverse this process. Catalbol enhances PGC-1α transcription via the cAMP/CREB pathway, upregulating mitochondrial genes (e.g., Tomm22 and ATP5d) and increasing oxygen consumption (109). Apple proanthocyanidins directly promote PGC-1α-mediated mitochondrial biogenesis, increasing mtDNA copy number and proteoglycan synthesis in OA mouse cartilage (110). Modulating the AMPK/SIRT1 energy-sensing network is another key strategy. In OA chondrocytes, decreased AMPKα phosphorylation leads to dysregulated energy sensing. Quercetin activates the AMPK/SIRT1 axis, increasing NRF1 nuclear translocation and inhibiting key glycolytic enzymes HK2 and LDHA, redirecting metabolism toward OXPHOS (111). Sestrin2 overexpression promotes mitochondrial biogenesis through AMPK/PGC-1α, increasing ATP production and reducing dorsal horn neuronal excitability in an OA pain model (112). Omentin-1, an adiponectin family member, upregulates TFAM via an AMPKα-dependent mechanism, increasing the mtDNA/nDNA ratio and improving respiratory chain complex activity in chondrocytes (113). Met induces SIRT3-dependent deacetylation of PGC-1α, synergistically activating NRF1/TFAM expression, which increases mtDNA copy number and enhances ATP synthase activity (114). β2-adrenergic receptor agonists like salbutamol inhibit the β-arrestin/GRK2 internalization pathway, upregulating PGC-1α expression and reducing MMP-13 activity, effectively delaying cartilage degradation (115) (**Figure 2**).

This figure illustrates the central role of mitochondrial dysfunction in OA and its therapeutic targets. The center of the image represents the affected joint, while the four surrounding quadrants illustrate four key pathological mechanisms: oxidative stress, metabolic reprogramming, dynamics imbalance, and mitophagy. These mechanisms are connected by arrows, forming an interaction network, with PGC-1α, Drp1, and PINK1-Parkin as key molecular targets.

### 3.5 Mitochondrial transfer therapy

Mitochondrial transfer therapy is an emerging regenerative strategy with revolutionary potential. Its core approach is to repair energy metabolism defects in damaged cells by replenishing exogenous functional mitochondria, thereby reversing core pathological processes (116). The theory stems from the natural phenomenon of mitochondrial transfer between cells. Studies have shown that bone marrow-derived mesenchymal stromal cells (BM-MSCs) can spontaneously transfer healthy mitochondria into impaired OA chondrocytes via a process mediated by connexin 43 (Cx43) and its isoform GJA1-20k (117). Chondrocytes that received MSC mitochondria were isolated by fluorescence-activated cell sorting technology, and their mitochondrial function was significantly restored: Δψm was improved (e.g., from 0.71 ± 0.12 to 1.79 ± 0.19), activity of respiratory chain complexes (I, II, III) and citrate synthase was enhanced, and intracellular ATP content was increased significantly (from 87.62 ± 11.07 nmol/mg to 161.90 ± 13.49 nmol/mg). This energy restoration is linked to fundamental improvements: the apoptosis rate of OA chondrocytes decreases (from 15.89% ± 1.30% to 7.09% ± 0.68%), and secretion of type II collagen (from 1.06 ± 0.11 to 2.01 ± 0.14) and aggrecan (from 0.97 ± 0.12 to 2.08 ± 0.20) increases approximately twofold (118). Notably, oxidative stress, a key causative factor in OA, actively triggers and enhances this mitochondrial donation, making transplantation more targeted in pathological microenvironments (117). Building on a deeper understanding of natural transfer mechanisms, artificial mitochondrial transfer (AMT) strategies have emerged to enable more direct and controlled therapeutic interventions (119). AMT involves isolating functional mitochondria from healthy donor cells (e.g., MSCs) and introducing them into damaged chondrocytes or tissues via techniques like electroporation, bioplasma carrier delivery, or direct injection. Experimentally, introduced MSC-derived mitochondria can successfully colonize and function in OA chondrocytes for up to nine days, restoring high ATP production and increasing the OXPHOS/glycolysis ratio, thereby reversing the pathological glycolytic shift. These exogenous mitochondria upregulate the fusion protein MFN2 and downregulate phosphorylated Drp1 (p- Drp1), reversing abnormal fragmentation. Importantly, AMT endows chondrocytes with robust resistance to oxidative damage by upregulating SOD2 transcription, expression, and activity, effectively scavenging excess ROS and reducing oxidative stress (90). This, combined with restored metabolic homeostasis, synergistically protects chondrocytes from apoptosis, significantly enhancing their survival in the harsh pathological microenvironment (90, 118).

The superiority of mitochondrial transplantation therapy is not only demonstrated by functional restoration at the cellular level but also demonstrated in animal models. In a collagenase-induced mouse OA model, direct intra-articular injection of healthy MSC-derived mitochondria reduced articular cartilage damage, improved histological scores, and helped maintain normal bone density (90). This directly demonstrates that transplanted mitochondria can continue to function protectively *in vivo*.

**Figure 3:**
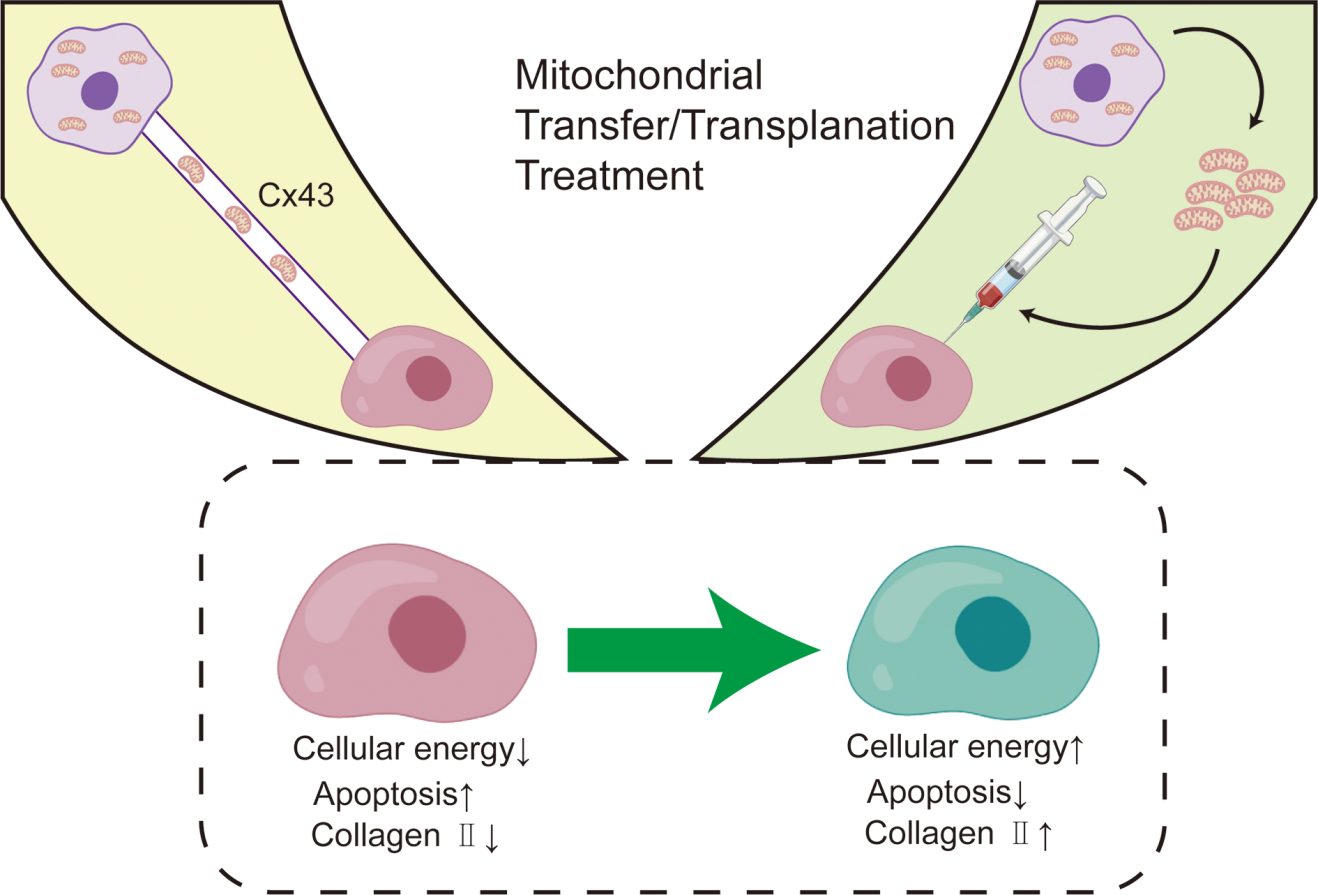
Mitochondrial transfer/transplantation treatment in OA.

Therefore, mitochondrial transfer therapy, whether based on natural mechanisms (117, 118) or AMT (119), represents a new paradigm that addresses the root causes of degenerative diseases such as OA. It moves beyond symptom relief to directly target core drivers: mitochondrial dysfunction and the resulting energy crisis, oxidative stress, and cell death. By replenishing functional mitochondria to restore energy homeostasis, optimize dynamics, enhance antioxidant defenses, and inhibit apoptosis (90), this therapy has the potential to halt or reverse cartilage degeneration, offering hope for a truly disease-modifying treatment for patients suffering from degenerative diseases such as OA. Despite its promising prospects, a comprehensive assessment of key safety and durability challenges is essential before clinical translation. A primary concern is the potential immunogenicity of allogeneic mitochondria. Furthermore, the fate and long-term survival of transplanted mitochondria within recipient chondrocytes remain not fully elucidated. Key questions include the stability of these exogenous organelles, their ability to replicate synchronously with the host cell cycle, and their ability to avoid selective clearance by mitochondrial quality control mechanisms such as mitophagy over extended periods. Addressing immunocompatibility and functional durability issues is crucial for developing safe and sustainable therapeutics (**Figure 3**).

### 3.6 Natural products and nutritional supplements

Natural products and nutritional supplements demonstrate significant potential for treating OA by improving mitochondrial function through multiple pathways. Melatonin activates the SIRT1/SOD2 axis, increasing chondrocyte Δψm, reducing ROS, promoting ATP synthesis, and enhancing ECM synthesis. Its sustained-release delivery system slowed cartilage degeneration in a rat post-traumatic OA model (78). Xanthohumol activates AMPK to enhance biogenesis while inhibiting the NLRP3 inflammasome and NF-κB pathways, attenuating palmitic acid-induced inflammation and matrix degradation (120). Hyaluronic acid protects mtDNA integrity: Pretreatment reduces oxidative stress-induced mtDNA damage, enhances repair capacity, and inhibits Cyt c release and apoptosis (76). Ginsenosides protect mitochondria synergistically. Ginsenoside Rg1 inhibits Bcl-2-associated X protein (Bax) activation, reduces Cyt c release, and decreases caspase-3 activity via the PI3K/Akt pathway, blocking IL-1β-induced apoptosis (121). Ginsenoside Rb1 stabilizes the mPTP, increases the B-cell lymphoma-extra large (Bcl-xL)/Bax ratio, and enhances chondrocyte survival under H_2_O_2_ stress (122). Low molecular weight xanthan gum inhibits chondrocyte apoptosis and reduces cytosolic Cyt c release by regulating the Bax-mitochondria-Cyt c-caspase pathway, maintaining cartilage matrix integrity in a rabbit anterior cruciate ligament transection model (123). These natural products offer a multi-target, low-toxicity therapeutic strategy for OA (**Table 1**).

**Table 1:** Summary of key studies on natural products improving mitochondrial function in OA.

Compound	Source	Key mechanisms	Main effects	Experimental model	PMID
Melatonin	pineal hormone	Activation of the SIRT1/SOD2 axis	Improve mitochondrial membrane potential, reduce ROS, and promote ATP synthesis and ECM production	Rat post-traumatic OA model	35726138
Xanthohumol	hops	Activate AMPK pathway and inhibit NLRP3/NF-κB	Enhances mitochondrial biogenesis, reduces inflammation and cartilage degradation	Human chondrocytes/high-fat diet mice	37964828
Hyaluronic acid	Connective tissue extraction	Protecting mtDNA integrity	Reduce mtDNA damage, enhance repair capacity, and inhibit apoptosis	Primary human chondrocytes	19193642
Ginsenoside Rg1	Ginseng	Regulates the PI3K/Akt/Bax-caspase pathway	Inhibits Cyt c release and caspase-3 activity, blocking mitochondrial apoptosis	Rat chondrocytes	24671491
Ginsenoside Rg1	Ginseng	Stabilize mitochondrial MPT	Increase the Bcl-xL/Bax ratio and enhance cell survival	Rat chondrocytes	23717124
Low molecular weight xanthan gum	microbial fermentation	Regulates the Bax-mitochondria-Cyt c apoptosis pathway	Inhibit chondrocyte apoptosis and reduce cytoplasmic Cyt c release	Rabbit ACLT model	None.

While these strategies have demonstrated significant efficacy in preclinical studies, most evidence comes from animal models or cell experiments, which differ from the chronic progression of autophagy in humans—for example, surgically induced OA models cannot fully simulate age-related mitochondrial decline. Secondly, there are risks of synergistic or conflicting effects between strategies: for instance, antioxidant therapy may interfere with normal ROS signaling, while autophagy activation requires precise regulation to avoid excessive clearance. Furthermore, while the multi-component nature of natural products is beneficial for multi-target interventions, standardization and bioavailability issues may limit clinical translation. Current research focuses too much on mechanism validation rather than the suitability for practical applications; for example, the impact of patient heterogeneity has not been fully explored.

## 4 Core challenges in clinical translation

The complex anatomy of joints poses significant barriers. The synovial membrane restricts the diffusion of macromolecules, the avascular cartilage matrix hinders deep drug penetration, and intracellular mitochondria require drugs to possess both cellular and organelle targeting capabilities (124). Traditional intra-articular injections struggle to maintain effective concentrations due to rapid clearance and low permeability. The development of novel, “intelligent” delivery systems is therefore urgently needed and represents a concrete research direction.​ For instance,​ nanocarriers can enhance cartilage targeting through surface modification with chondrocyte-affinity peptides or ligands for receptors overexpressed in OA tissues; smart hydrogels enable sustained, responsive drug release triggered by specific joint microenvironment cues such as pH, enzyme activity, or reactive oxygen species levels; and exosomes, with natural homing ability and low immunogenicity, hold promise as carriers for mitochondrial components or drugs and can be further engineered for enhanced targeting. Furthermore, treatments targeting chondrocyte mitochondria require additional​ mitochondrial targeting design, often achieved by conjugating drugs to lipophilic cations or mitochondria-penetrating peptides (125). However, achieving efficient cartilage penetration and subsequent​ precise mitochondrial delivery remains an unresolved technical bottleneck that requires interdisciplinary innovation in materials science and bioengineering.

OA is a heterogeneous disease affecting multiple joint tissues (cartilage, synovium, subchondral bone). Mitochondrial dysfunction patterns vary: Chondrocytes:face impaired energy metabolism, while synovial cells are more susceptible to inflammation-related dysregulation. Furthermore, patients have diverse etiologies (mechanical injury, metabolic abnormalities, aging), varying disease stages, and molecular phenotypes. Consequently, single-target interventions may only be effective in specific subpopulations. For example, mitophagy strategies may benefit age-related OA but have limited efficacy in traumatic OA. Therefore, biomarker-based patient stratification and personalized combination treatments are necessary (126).

Existing OA models fail to fully recapitulate the human disease. Surgically induced models simulate acute mechanical injury, whereas human OA is often chronic and progressive. Chemically induced models create intense inflammation, inconsistent with progressive mitochondrial damage in primary OA. Most experiments use young animals, ignoring the impact of aging, a core risk factor for OA, on mitochondria. These limitations reduce the reliability of preclinical data for predicting efficacy, necessitating more human-like models (such as humanized organoids and aging-induced models) to enhance translational value (127, 128).

As the cellular energy hub, mitochondria require vigilant risk assessment for targeted interventions. Mitochondria-targeted antioxidants may interfere with normal redox signaling; overactivation of autophagy may excessively clear healthy mitochondria; suppressing ROS to subphysiological levels may impair cellular defense mechanisms. Most mitochondrial drugs lack long-term clinical monitoring, and systemic administration may affect high-energy-consuming organs (e.g., heart and brain) (129). Therefore, rigorous preclinical toxicology evaluation and long-term post-marketing follow-up are necessary to balance therapeutic benefits against potential risks.

In summary, the clinical translation of mitochondrial-targeted therapies requires a coordinated effort to overcome delivery technology bottlenecks, address disease heterogeneity, optimize disease models, and establish a long-term safety monitoring system to achieve the transition from basic research to clinical application.

## 5 Conclusion

Mitochondrial dysfunction has been identified as a core driver of OA pathogenesis, accelerating cartilage degeneration by triggering an energy crisis, oxidative stress imbalance, and cell death cascade. Targeting mitochondrial function restoration—encompassing antioxidant defense, dynamics balance, autophagy activation, and biosynthesis regulation—has emerged as a promising new therapeutic paradigm. Although obstacles in delivery, heterogeneity, and model translation remain, interdisciplinary collaboration and technological integration will bring transformative treatment hope to hundreds of millions of patients with OA.
